# Gold nanoparticles in skin drug delivery: a bibliometric analysis of research trends from 2015 to 2025

**DOI:** 10.3389/fmedt.2026.1808952

**Published:** 2026-06-03

**Authors:** Mohammed Arif, Karim Mousa, Haider Butt

**Affiliations:** 1 Department of Mechanical & Nuclear Engineering, Khalifa University of Science and Technology, Abu Dhabi, United Arab Emirates; 2 Advanced Digital & Additive Manufacturing (ADAM) Center, Khalifa University of Science and Technology, Abu Dhabi, United Arab Emirates

**Keywords:** bibliometric analysis, gold nanoparticles, nanocarrier, skin drug delivery, topical drug delivery

## Abstract

Gold nanoparticles (AuNPs) have emerged as highly effective nanocarriers for skin drug delivery due to their tunable physicochemical properties, large surface area, and ease of functionalization. Over the past decade, research in this field has expanded rapidly, driven by advances in nanotechnology and increasing interest in non-invasive topical and transdermal-based therapies. In this study, a comprehensive bibliometric analysis was conducted to evaluate global research trends in gold nanoparticle-based skin drug delivery between January 1, 2015, to December 31, 2025. using the Web of Science and Scopus databases. Publications were identified through structured keyword-based searches focused on “gold nanoparticles” and “skin drug delivery”, and analyzed for publication output, leading contributors, research areas, and keyword co-occurrence using VOSviewer. The results reveal a significant growth in publications, particularly after 2020, with major contributions from Asia, especially India and China. Key research themes include wound healing, antibacterial applications, and skin cancer treatment, with an increasing emphasis on nanocarrier design and controlled drug-delivery systems. This study provides a systematic overview of the research landscape, offering insights into emerging trends and influential contributors. However, it is limited by database selection, potential keyword bias, and the descriptive nature of bibliometric analysis, which does not include experimental validation. Despite these limitations, the findings contribute to a clearer understanding of the development and future direction of gold nanoparticle-based skin drug delivery research.

## 1 Introduction

Nanoparticle-based drug delivery systems have attracted significant attention in recent years, particularly for topical and transdermal applications. A wide range of nanocarriers, including polymeric nanoparticles (1), lipid-based systems such as liposomes and solid lipid nanoparticles (2), and metallic nanoparticles (3), have been explored to enhance drug stability, improve skin penetration, and enable controlled release. Each class offers distinct advantages depending on the therapeutic need, such as biocompatibility in lipid systems or structural tunability in polymeric carriers. These nanosystems are especially important for overcoming the barrier function of the stratum corneum, which limits the effectiveness of conventional drug-delivery approaches (4). Among these, metallic nanoparticles, especially gold nanoparticles (AuNPs), are promising due to their unique physicochemical properties and multifunctional nature.

Gold nanoparticles (AuNPs) are nanomaterials measuring 1–100 nm, which have gained immense interest for several applications in the biomedical field, especially because they possess unique physicochemical properties such as a high surface area-to-volume ratio, the ability to be designed for various sizes and shapes, surface plasmon resonance, and easy functionalization. These characteristics make the interaction with biological systems easy, and they are of high importance in order to increase the efficiency of delivering drugs (5–7). AuNPs have been widely investigated for their use as carriers to enhance drug permeation through the stratum corneum, as protective agents to prevent degradation of active pharmaceuticals, and as means for controlled drug release (8, 9).

Gold nanoparticles can be produced using various methods and functionalized with polymers, ligands, or therapeutic molecules to enhance the stability, biocompatibility, as well as targeting efficiency (10). The AuNPs have relatively low cytotoxicity and biocompatibility, making them more appropriate for topical or transdermal applications, such as the treatment of inflammatory dermatitis (11), infections (4), and cancer (12), as well as for cosmetic applications (13). Also, gold nanoparticles exhibit photothermal as well as antimicrobial properties, further expanding their therapeutic potential in dermatology and oncology (14).

Recent breakthroughs have demonstrated the potential of gold nanoparticles dispersed through a variety of drug delivery systems, including creams, gels, microneedles, and hydrogels for sustained delivery of drugs through the skin (15, 16). Such a hybrid system shows improved drug retention near the target. In addition, the AuNP-based system has shown significant potential for imaging and diagnostic applications, providing contrast enhancement. Such properties will provide much assistance in theranostic applications (17, 18).

Over the past decade, studies relating to the use of gold nanoparticles in drug delivery to the skin have increased at a rapid pace, and this is fuelled by technological advances (19) and by interdisciplinary collaboration between pharmaceutical scientists, materials scientists and clinicians (20). However, despite the growing body of literature, there is a lack of systematic evaluation of trends in publications, hotspots, and contributions from around the globe. Therefore, this study aims to analyse the growth and evolution of research on gold nanoparticles in skin drug delivery through a bibliometric analysis of publications indexed in the Web of Science (WOS) and Scopus databases from January 1, 2015, to December 31, 2025. This analysis gives us an idea of the trends in research, the most important countries, the most important writers, and the new issues that are coming up in this quickly changing sector.

## 2 Methodology

Several commonly used databases, such as PubMed, Google Scholar, Embase, and ScienceDirect, were explored because they cover a wide range of scientific literature. But for bibliometric work, simple coverage is not enough. What matters more is how well the data is organized and how reliably citations can be tracked. In this regard, WOS and Scopus offer more structured search options, cleaner metadata, and stronger citation analysis tools. Because of this, Scopus and WOS were chosen to ensure consistent, reproducible, and complete data collection.

This bibliometric analysis looked at the Web of Science (WOS) and Scopus databases to see what research has been done on using gold nanoparticles to deliver drugs through the skin. You can do advanced searches by topic in both databases, which means you can use structured queries to find publications that are very relevant. The advanced search strategy concentrated on locating the terms “gold nanoparticles” and “skin drug delivery” within the titles, abstracts, or keywords of publications, encompassing author keywords, indexed keywords, and Keywords Plus, to ensure consistency across searches in both databases. The query was changed to include different forms and combinations of the search terms, such as singular and plural forms, abbreviations (like “AuNPs”), and related phrases like “transdermal drug delivery” and “topical drug delivery.” These terms focus on both the nanomaterial and its application, helping to avoid unrelated studies. Although broader terms such as “topical nanomedicine” or “transdermal nanocarriers” were considered, they were not used as primary search terms because they might yield a broader range of publications not specifically related to gold nanoparticles. However, related terms such as “transdermal drug delivery” and “topical drug delivery” were also included to make the search more comprehensive.

The “AND” operator was used in the title, abstract, or keyword search fields to make sure that both ideas were covered in the same publication. The “OR” operator was not used because it could have found publications that talked about gold nanoparticles or skin drug delivery separately. To get the most up-to-date research trends and developments in the field, the search results were narrowed down even more by limiting the publication years to 2015 to 2025. The starting year 2015 was selected based on the observed increase in publication output, indicating the beginning of rapid research expansion in this field. The inclusion of 2025 ensures coverage of the most recent trends, although data for this year remain subject to ongoing database updates.

Information relevant to the bibliography, such as publication year, authors, affiliations, countries, document types, and citation counts, was extracted by both databases, and later a quantitative analysis was performed. The data were plotted and analysed using Origin to visualize publication trends and citation patterns over time. It is worth noting that the figures used in this research are the most accurate approximations; it is impossible to be sure, as the number of publications, citations, and indexing is expected to grow, especially by 2025. Furthermore, data from WOS and Scopus were reported separately to avoid duplication, as several publications are indexed in both databases.

Several tools are available for bibliometric analysis, including VOSviewer, CiteSpace, BibExcel, and Gephi. Each tool has its own strengths. For example, CiteSpace is useful for identifying emerging trends and citation bursts, while Gephi offers more flexibility for network customization and visualization. In this study, VOSviewer was selected because it is easy to use and can efficiently handle large datasets. It is particularly suitable for creating and visualizing co-authorship, co-citation, and keyword co-occurrence networks. It also uses a consistent clustering method that helps in clearly identifying research themes and relationships. However, it has some limitations, such as fewer customization options compared to Gephi and less focus on time-based analysis than CiteSpace. Overall, its balance of simplicity, performance, and clear visualization makes it appropriate for this bibliometric study on gold nanoparticle-based skin drug delivery.

## 3 Results and discussion

The results and discussion section presents a comprehensive bibliometric analysis of research on gold nanoparticles for skin drug delivery, based on data from the WOS and Scopus databases. The findings are organized into several subsections, including publication trends over time, distribution of document types, contributions by countries and funding agencies, leading journals and institutions, and analysis of highly cited and recent publications. In addition, keyword co-occurrence analysis is used to identify major research themes and emerging topics within the field. This structured approach provides a clear overview of the development, current status, and research directions in this area.

### 3.1 Literature publication growth

This subsection examines the annual publication trends to highlight the growth and evolution of research activity in this field.

**Figure 1:**
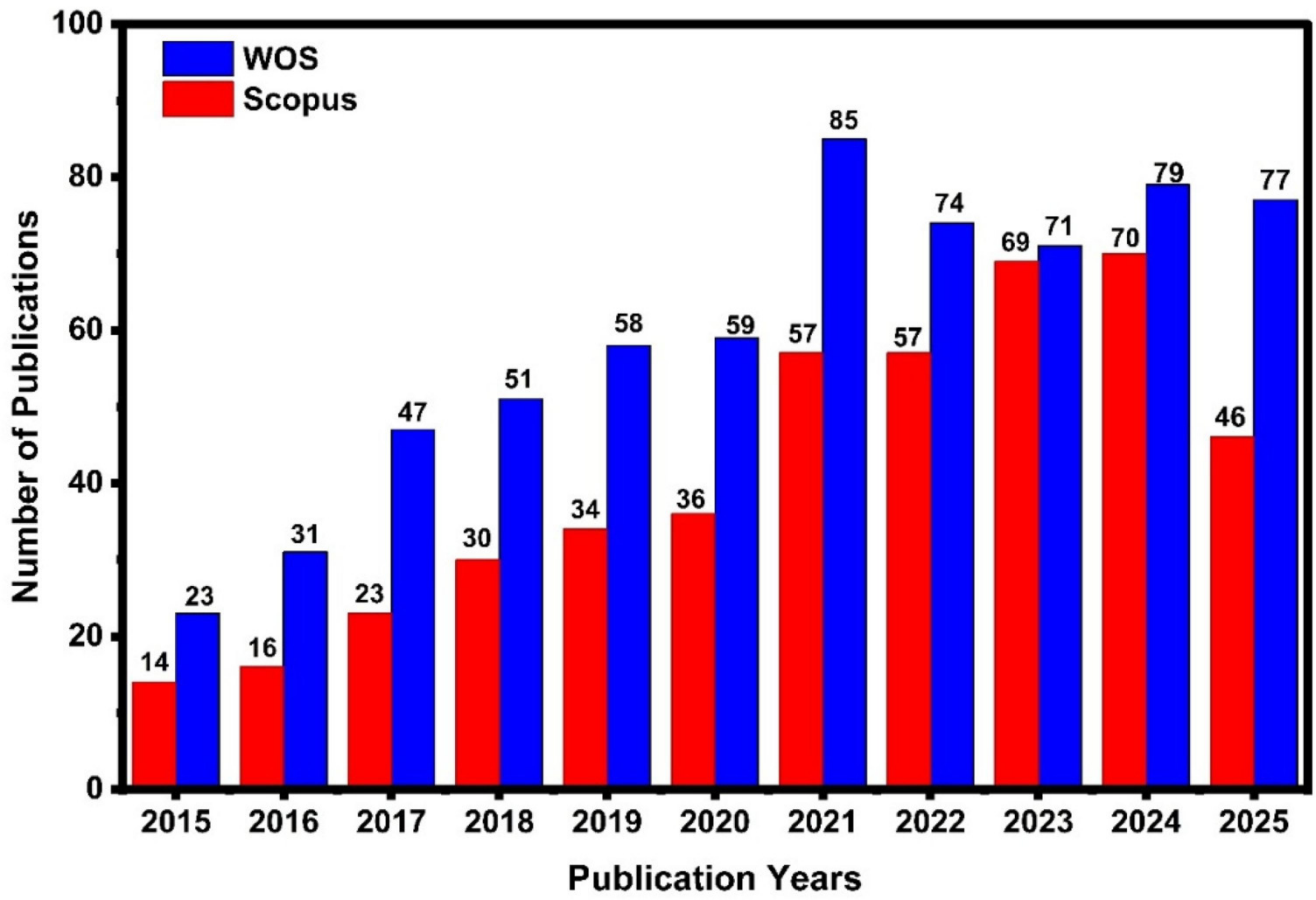
Number of publications per year (from 1 January 2015 to 31 December 2025.

The retrieved data from the Scopus and Web of Science (WOS) databases showed that the number of publications on gold nanoparticles in skin drug delivery varied, with WOS reporting more publications than Scopus. This variation can be explained by differences in database coverage, indexing policies, and journal inclusion criteria. The advanced search strategy was implemented in both databases to reduce the differences and identify and select articles that address the keywords “gold nanoparticles”, “skin drug delivery”, and their variants in the title, abstract, and author keywords. The recovered materials were slowly filtered to retain the relevant material, and unnecessary records were disregarded. The bibliometric analysis, as presented in Figure 1, comprises the selected publication types published between 1 January 2015 and 31 December 2025. The databases were consistent and comparable, so no publications published before 2015 were included. The largest number of publications in WOS was in 2021, with 85, and in Scopus, the best publication output was in 2024, with 70. In general, both databases show an evident and steady rise in research output during the period of the study, especially since 2020, which can be attributed to growing interest in the field and increased research activity on gold nanoparticle-based skin drug delivery systems. This sharp increase after 2020 coincides with rapid advancements in nanotechnology based transdermal systems, particularly microneedle based delivery, photothermal therapy, and multifunctional hydrogel platforms, which have expanded the applicability of gold nanoparticles in dermatology.

**Figure 2:**
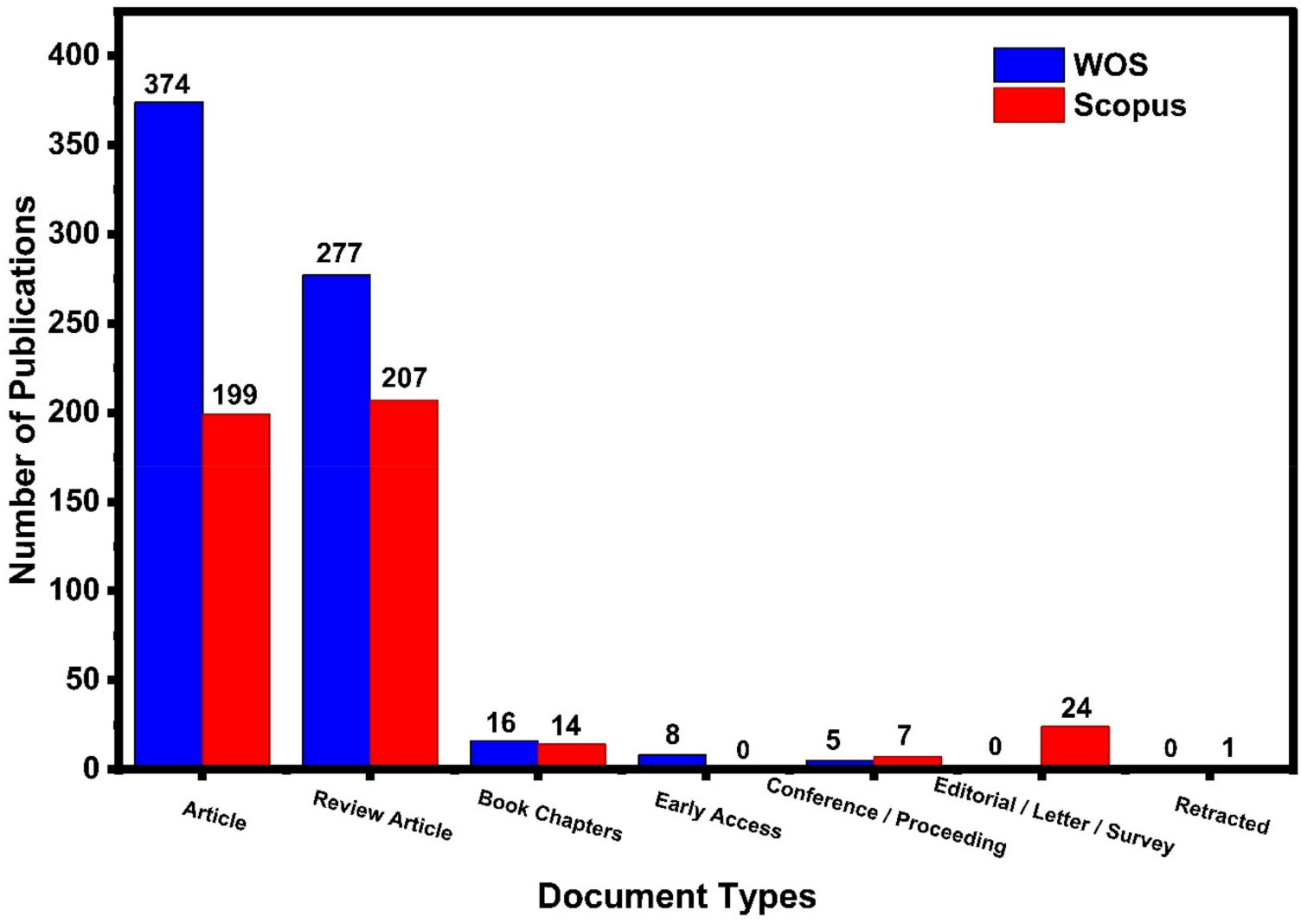
Number of published document types.

Figure 2 presents the different publication types indexed in the Web of Science (WOS) and Scopus databases. The publications included in this bibliometric analysis are articles, review articles, book chapters, early access publications, conference proceedings, editorials/letters/surveys, and retracted publications, though preprints were not included. The publications considered were limited to those that used precise or closely related terminology in both databases; hence, the zero values might indicate variations in database classification, rather than the inability to find any publications.

In both databases, the predominant document type is original research articles. Articles account for 374 publications in the WOS database, about 55.00% of the total number of records, and the remaining records are review articles (277 publications). Compared with Scopus, the database documents 199 research articles (44.03%) and 207 review articles (45.80%), indicating a relatively high ratio of review articles. Other document types, such as book chapters, proceedings, early access papers, editorials, and retracted documents, have a slight effect on the total number of publications in both databases.

As shown in Figure 2, Scopus indexes other document types like editorials, letters, and surveys that are either underrepresented or non-existent in WOS, which in part might be the underlying cause of the differences in the total number of publications. In general, the prevalence of research articles and reviews is associated with the intensive focus on experimental research and detailed synthesis in the sphere of gold nanoparticle-based skin drug delivery, and the low number of conference proceedings, clinical translations, and patents indicate that the translation and practical advancement is underrepresented in the research area considered during the period of the study, between 2015 and 2025.

**Figure 3:**
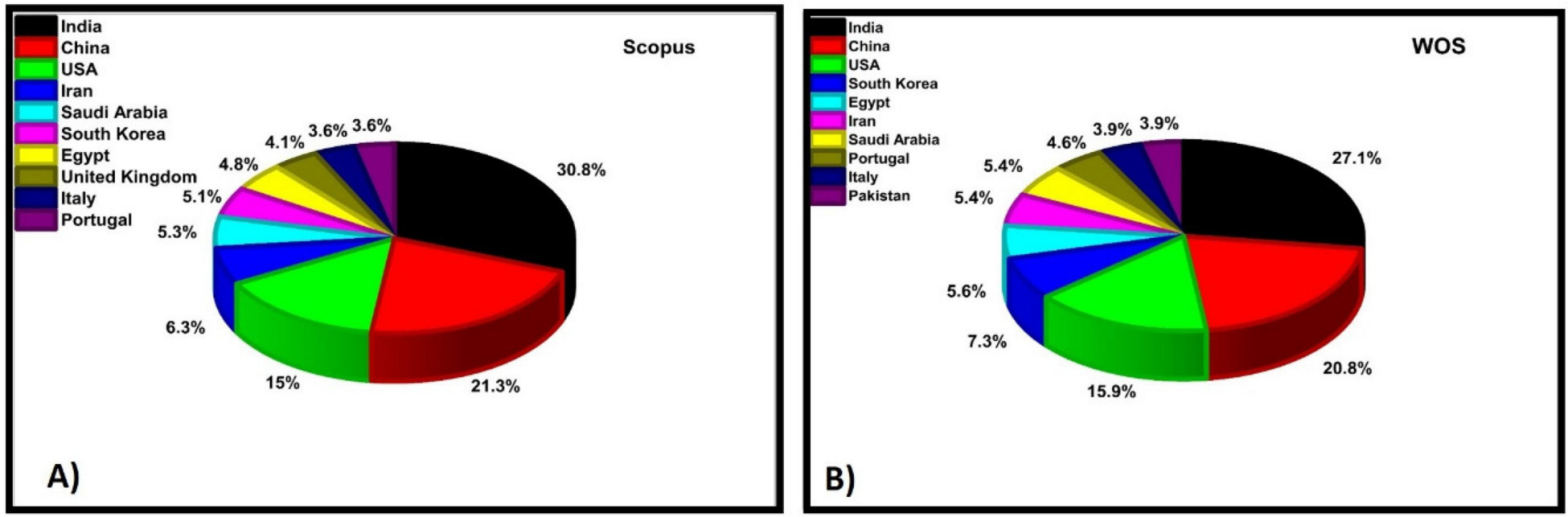
Top 10 countries with the most publications: **(A)** Scopus, **(B)** WOS.

As shown in Figure 3, this field attracts international attention. Both the Scopus and the Web of Science (WOS) databases show that India and China have become the top contenders. The dominance of China and India is not only quantitative but also reflects strong national funding support and institutional research capacity, particularly in nanotechnology and pharmaceutical sciences, which have accelerated innovation in gold nanoparticle based drug delivery systems. This dominance is strongly influenced by national funding support, particularly from agencies such as the National Natural Science Foundation of China, as well as growing institutional research capacity and strategic focus on nanotechnology and drug delivery systems. The United States of America (15.0%), China (21.3%), and India (30.8%) constitute the largest share of publications in the Scopus database. Likewise, in the WOS database, India has the highest share (27.1%) of all publications, followed by China (20.8%) and the USA (15.9%). Other major contributors to both databases are South Korea, Iran, Saudi Arabia, and Egypt, with between 5 and 7 percent contribution to the total research output, respectively. The other nations on the top ten list have smaller proportions, typically less than 5%. Italy and Pakistan are among the lower-ranked countries within the top ten contributors in the WOS database, each accounting for approximately 3.9% of total publications. In comparison, Portugal and Italy appear among the lower-ranked countries in the Scopus database, each contributing nearly 3.6% of the total publications. In general, the distribution by country level shows that most of the research on the topic of gold nanoparticle-based skin drug delivery is done by the Asian countries, with significant contributions made by North America and Europe, which is reflective of the research priorities in the region, as well as the growing global involvement in nanotechnology-based research on dermatological drug delivery research over the time period between 2015 and 2025. Differences in international collaboration patterns and database coverage may also contribute to the observed regional distribution.

Tables 1, 2 summarise the top 10 funding agencies extracted from WOS and Scopus, respectively. A few agencies are listed in both databases as top contributors, suggesting overlap in funding priorities across regions. The most prominent funding agency in the WOS database is the National Natural Science Foundation of China (NSFC), which funds 74 publications, accounting for about 27.8 percent of the total publications funded by the top 10 funding agencies, as shown in Table 1. The United States has the most notable funding organizations, namely the National Institutes of Health (NIH) and the United States Department of Health and Human Services (HHS), which together contributed 36 publications (13.5% and 13.5%, respectively). Other significant sources are the Instituto Politécnico Nacional (Mexico) and the National Research Foundation of Korea. The last-ranked agencies on the WOS top 10 list are the Conselho Nacional de Desenvolvimento Científico e Tecnológico (CNPq) of Brazil and the European Research Council (ERC), which each fund 12 publications (about 4.5%). The WOS data, in general, indicate that the major funding agencies, Chinese and U.S., are the ones that promote research on skin drug delivery based on gold nanoparticles.

**Table 1:** Top 10 funding agencies according to WOS.

No.	Funding agencies	Country/region	Number of publications
1	National Natural Science Foundation of China	China	74
2	National Institutes of Health (NIH)	United States	36
3	United States Department of Health and Human Services (HHS)	United States	36
4	Instituto Politécnico Nacional	Mexico	32
5	National Research Foundation of Korea	South Korea	18
6	National Institute of Arthritis and Musculoskeletal and Skin Diseases (NIAMS)	United States	17
7	Fundação para a Ciência e a Tecnologia (FCT)	Portugal	15
8	National Science Foundation (NSF)	United States	14
9	Conselho Nacional de Desenvolvimento Científico e Tecnológico (CNPq)	Brazil	12
10	European Research Council (ERC)	European Union	12

**Table 2:** Top 10 funding agencies according to Scopus.

No.	Funding agencies	Country/region	Number of publications
1	National Natural Science Foundation of China	China	47
2	National Institutes of Health	United States	13
3	Fundação para a Ciência e a Tecnologia	Portugal	11
4	European Commission	European Union	10
5	National Key Research and Development Program of China	China	10
6	National Research Foundation of Korea	South Korea	10
7	National Cancer Institute	United States	9
8	Department of Science and Technology, Ministry of Science and Technology	India	8
9	National Science Foundation	United States	6
10	Conselho Nacional de Desenvolvimento Científico e Tecnológico	Brazil	5

Similarly, the Scopus database shows that the leading funding agency is the National Natural Science Foundation of China, with 47 publications, accounting for about 36.4 per cent of all publications from the top 10 agencies (as shown in Table 2). The top U.S. funding agency in Scopus is the National Institutes of Health, with 13 publications (approximately 10.1%), followed by the National Cancer Institute and the National Science Foundation. Other agencies with wider international involvement include the European Commission and Fundacao para a Ciencia e a Tecnologia (FCT), both of which are among the top 10 agencies. Compared with WOS, Scopus shows a slightly more diffuse funding landscape across regions. However, both databases highlight the dominance of Chinese funding agencies, which have driven significant investment and interest in research on the application of gold nanoparticles for the delivery of skin drugs. The presence of highly cited publications from these regions suggests that their contribution is not only quantitative but also reflects increasing scientific impact in the field. The lower number of funded publications in Scopus compared to WOS may be due to differences in how the databases are built. Scopus covers a wider range of journals, including many that do not always report funding details. In contrast, WOS includes more selectively indexed journals, which often follow more consistent reporting practices. Because of this, funding information is more clearly captured in WOS. Also, the broader coverage in Scopus may reduce the visibility of funding data, leading to lower reported counts of funded publications.

### 3.2 Paper publication

This subsection analyses the distribution of publication types to understand the nature and composition of the research output.

Table 3 shows the most published articles on gold nanoparticles in skin drug delivery that rank highest in the WOS database and Scopus database as per 1 January 2015 to 31 December 2025. When comparing the two databases, it can be determined that a number of journals are ranked high in both WOS and Scopus, with a difference in the number of publications and relative ranking. The Journal of Drug Delivery Science and Technology is the first publication with the highest number of publications, with 32 publications, corresponding to about 24.8% of the total number of publications in the top-ranked WOS journals. These are then the International Journal of Pharmaceutics and Pharmaceutics, ranked second with 25 publications (around 19.4% each). However, the Journal of Drug Delivery Science and Technology is also first in the Scopus database with 32 publications, which is approximately 27.9 percent of the total publications in the top Scopus journals, compared to Pharmaceutics with 27 publications (23.5%).

**Table 3:** Top journals with the highest number of publications.

WOS		Scopus	
Publication title (Source)	Number of publications	Publication title (Source)	Number of publications
Journal of Drug Delivery Science and Technology	32	Journal of Drug Delivery Science and Technology	32
International Journal of Pharmaceutics	25	Pharmaceutics	27
Pharmaceutics	25	International Journal of Nanomedicine	15
ACS Applied Materials & Interfaces	14	International Journal of Biological Macromolecules	12
Journal of Controlled Release	14	Advanced Drug Delivery Reviews	11
Colloids and Surfaces B: Biointerfaces	11	Artificial Cells Nanomedicine and Biotechnology	9
International Journal of Molecular Sciences	11	Current Pharmaceutical Design	8

Other journals like the International Journal of Nanomedicine and the International Journal of Biological Macromolecules are in the mid-range in Scopus but not in the top list of the WOS, which also demonstrates differences in coverage in the databases. On the other hand, some journals, such as ACS Applied Materials and Interfaces and Journal of Controlled Releases, are ranked among the top sources in WOS but not among the top Scopus journals. The last in the WOS top list are Colloids and Surfaces B: Biointerfaces and the International Journal of Molecular Sciences, which have 11 publications (around 8.50%). In Scopus, the lowest position is held by Current Pharmaceutical Design, with 8 publications (around 7.00%). Overall, although both databases report the same main sources of publications, which include drug delivery, pharmaceutics, and nanomedicine journals as the major ones, the differences in ranking and inclusion of journals also add to the existing disparity in the distribution of publications between WOS and Scopus.

**Figure 4:**
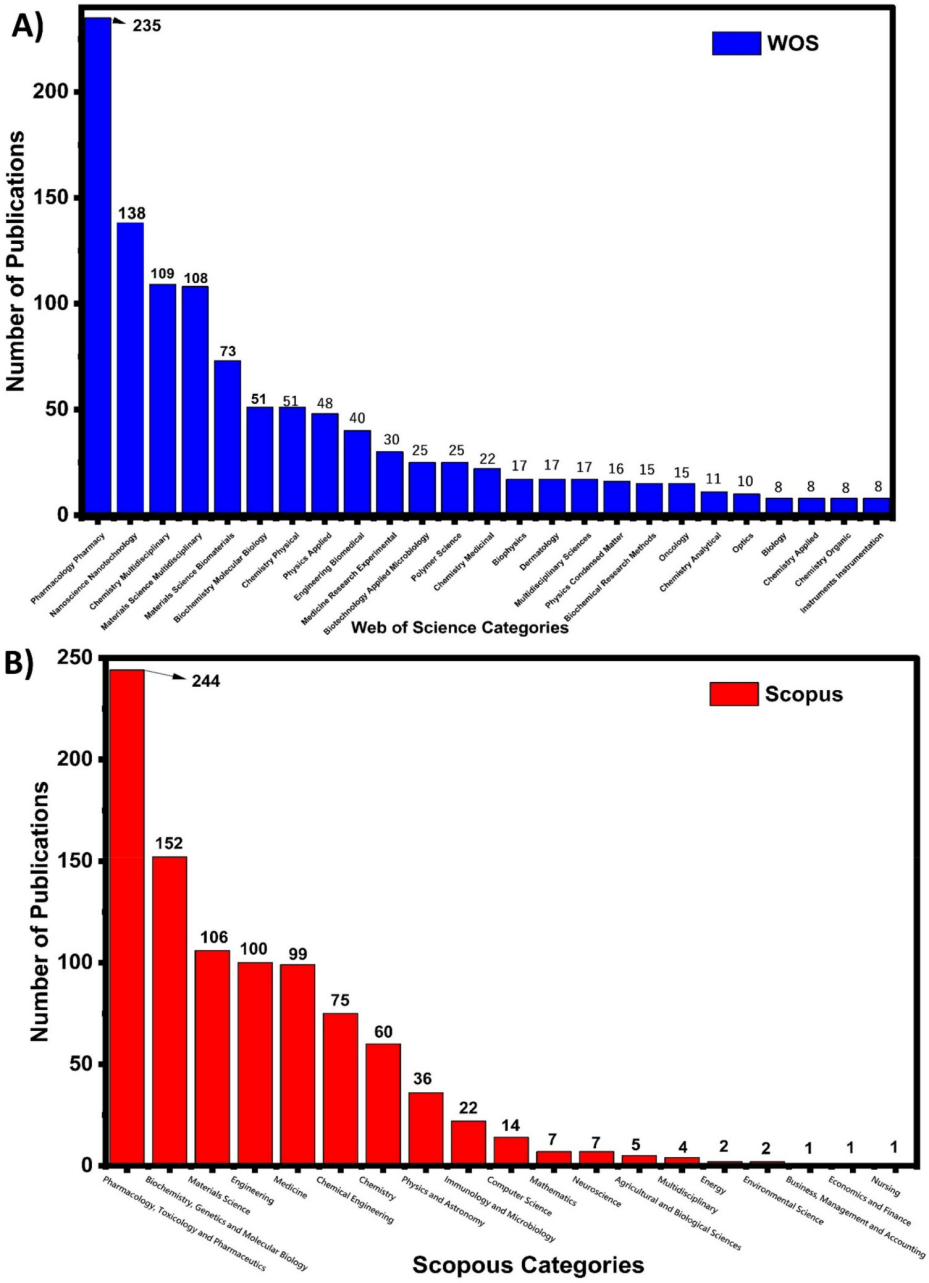
Number of publications based on the research area according to: **(A)** WOS, **(B)** Scopus.

The Web of Science and Scopus databases classify publications into multiple research categories, with Web of Science using more specific subject classifications and Scopus applying broader disciplinary groupings, as demonstrated in Figures 4A,B. Figure 4B shows that Pharmacology, Toxicology, and Pharmaceutics is the leading Scopus category with 244 publications, followed by Biochemistry, Genetics, and Molecular Biology with 152 publications and Materials Science with 106 publications, together representing nearly two-thirds of the total Scopus output. Engineering and Medicine also contribute substantially to Scopus, with 100 and 99 publications, respectively. Figure 4A indicates that Pharmacology and Pharmacy dominate the Web of Science categories with 235 publications, followed by Nanoscience and Nanotechnology with 138 publications and Chemistry, Multidisciplinary with 109 publications, accounting for more than half of the total Web of Science records. Materials Science-related categories, including multidisciplinary materials science and biomaterials, further reinforce the field's strong materials-driven research focus. Overall, Figures 4A,B clearly demonstrate that research on gold nanoparticles in skin drug delivery from 2015 to 2025 is concentrated primarily in pharmacology, materials science, and nanotechnology, while contributions from peripheral disciplines remain limited.

**Figure 5:**
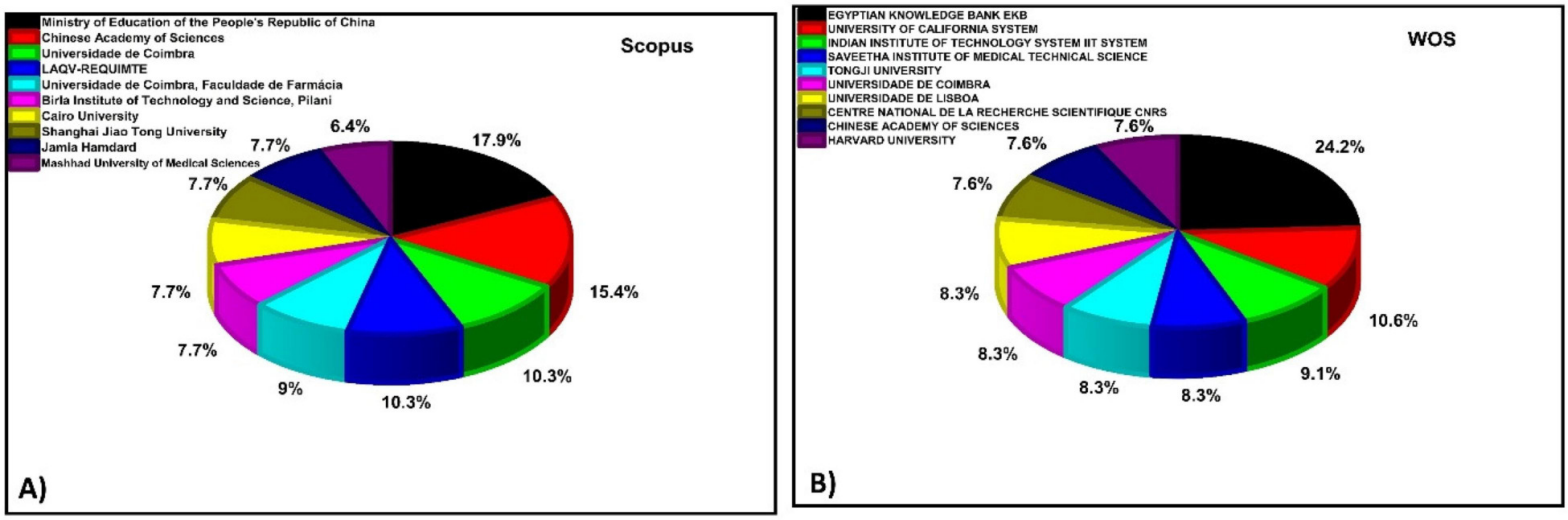
Top 10 organizations listed with the highest number of publications according to: **(A)** WOS, **(B)** Scopus.

Figure 5 shows the top ten affiliated organizations with the highest number of publications on gold nanoparticles in skin drug delivery according to the WOS and Scopus databases. According to WOS (Figure 5A), the Egyptian Knowledge Bank ranks first with a contribution of 24.2% of the total publications, followed by the University of California system at 10.6% and the Indian Institute of Technology system at 9.1%, while the remaining leading institutions each contribute between approximately 7.6% and 8.3%. In contrast, the Scopus database (Figure 5B) shows that the Ministry of Education of the People's Republic of China leads with 17.9%, followed by the Chinese Academy of Sciences at 15.4%, and the Universidade de Coimbra and Shanghai Jiao Tong University with contributions of around 10.3% and 9.0%, respectively. Although several organizations are common between the two databases, differences in percentage contributions and ranking order highlight the impact of database coverage on institutional bibliometric outcomes.

### 3.3 Preferred publications

This subsection identifies the leading journals to highlight the primary publication sources in this research area. Table 4 presents a summary of the top 10 most cited publications related to gold nanoparticles in skin drug delivery identified from the WOS and Scopus databases between 2015 and 2025. There are more research articles than review articles in these publications, which shows that this field is very experimental. The thermoresponsive gold nanoparticle-based transdermal antibacterial gel for burn wound treatment by Arafa et al. (21), published in Scientific Reports in 2018, is the most cited article in both databases. It was cited 178 times in WOS and 231 times in Scopus. Several other highly cited articles provide further insight into the functional role of gold nanoparticles in skin drug delivery. For example, Gupta et al. (22) demonstrated that nanoparticle size and surface charge significantly influence skin permeability, highlighting the importance of physicochemical properties in transdermal transport. Similarly, Casciaro et al. (23) showed that peptide-functionalized gold nanoparticles enhance antimicrobial activity and promote wound healing, emphasizing their therapeutic potential. Furthermore, Xu et al. (25) reported a photothermal system capable of simultaneous antibacterial action and tissue regeneration, while Bessar et al. (28) developed a nanoparticle-based system for improved topical drug delivery in psoriasis. These studies collectively illustrate the diversity of applications and reinforce the strong experimental focus seen across both WOS and Scopus datasets. These studies also highlight the importance of nanoparticle size, surface charge, and hydrophobicity in enhancing penetration through the stratum corneum, which directly influences drug delivery efficiency. Also, highly cited studies highlight a shift from basic nanoparticle characterization toward application-driven research, particularly in enhancing skin permeability, antimicrobial activity, and targeted cancer therapy.

**Table 4:** Summary of the top 10 cited publications per database.

Article title	Article type	Authors	Journal name	Country	Summary	Year	Citations (WOS/Scopus)		Reference
Thermoresponsive gels containing gold nanoparticles as smart antibacterial and wound healing agents	Research Article	Arafa et al.	SCIENTIFIC REPORTS	Egypt	A thermoresponsive gold nanoparticle drug delivery gel for the treatment of burn wounds was developed. The formulation exhibited suitable gelation behaviour, antibacterial activity against Staphylococcus aureus, and enhanced wound healing ability *in vivo*, indicating its potential as an effective transdermal antibacterial delivery system.	2018	178	231	(21)
Effect of Size and Surface Charge of Gold Nanoparticles on their Skin Permeability: A Molecular Dynamics Study	Research Article	Gupta et al.	SCIENTIFIC REPORTS	India	The research demonstrates that neutral hydrophobic gold nanoparticles can infiltrate and disrupt skin lipid membranes, whereas charged nanoparticles remain on the surface. These insights help us design nanoparticles that can be used to deliver drugs through the skin and for cosmetic purposes.	2017	166	191	(22)
Gold-nanoparticles coated with the antimicrobial peptide esculentin-1a-(1-21)NH₂ as a reliable strategy for antipseudomonal drugs	Research Article	Casciaro et al.	ACTA BIOMATERIALIA	Italy	This study demonstrates that conjugating the antimicrobial peptide Esc(1-21) to gold nanoparticles (AuNPs) greatly enhances its stability, antibacterial activity against Pseudomonas aeruginosa, and safety for human skin cells. The peptide-coated AuNPs also promote wound healing, highlighting their potential for skin-targeted drug delivery and topical treatment of infections.	2017	148	168	(23)
Therapeutic Applications of Curcumin Nanoformulations	Review Article	Yallapu et al.	AAPS Journal	USA	This review highlights curcumin's broad therapeutic potential but emphasizes that its clinical use is limited by poor solubility, stability, and bioavailability. It discusses how curcumin nanoformulations improve pharmacokinetics and targeted drug delivery, summarizes current clinical evidence, and outlines prospects for curcumin-based pharmaceutical and nutraceutical applications.	2015	354		(24)
Controlled-temperature photothermal and oxidative bacteria killing and acceleration of wound healing by polydopamine-assisted Au-hydroxyapatite nanorods	Research Article	Xu et al.	Acta Biomaterialia	China	This study presents a gold nanoparticle-based photocatalytic system that integrates antibacterial properties and skin wound healing through the generation of hydroxyl radicals and mild heat under near-infrared irradiation. The system quickly kills bacteria without hurting tissue and at the same time encourages tissue regeneration. This shows that it could be a safe and effective way to treat skin wounds and deliver drugs with nanoparticles.	2018	217		(25)
Biologically synthesized green gold nanoparticles from Siberian ginseng induce growth-inhibitory effect on melanoma cells (B16)	Research Article	Wu et al.	Artificial Cells, Nanomedicine and Biotechnology	China	This study presents the environmentally friendly synthesis of gold nanoparticles utilizing Siberian ginseng extract and illustrates their anticancer efficacy against melanoma, a formidable skin malignancy. The biosynthesized gold nanoparticles caused oxidative stress and apoptosis in melanoma cells, showing that they could be useful for treating skin cancer and delivering drugs through nanoparticles.	2019	215		(26)
Challenges and Future Prospects for the Delivery of Biologics: Oral Mucosal, Pulmonary, and Transdermal Routes	Review Article	Morales et al.	AAPS Journal	USA	This review discusses the key challenges in delivering biologic drugs through oral mucosal, pulmonary, and transdermal routes, including biological barriers, stability issues, and limited absorption. It also highlights emerging strategies and prospects in drug delivery systems to improve the safe and effective transport of biologics across skin and other non-invasive routes.	2017	143		(27)
Functionalized gold nanoparticles for topical delivery of methotrexate for the possible treatment of psoriasis	Research Article	Bessar et al.	Colloids and Surfaces B: Biointerfaces	Egypt	This study developed a gold nanoparticle based topical drug delivery system to enhance the skin penetration, stability, and efficacy of methotrexate for psoriasis treatment. Hydrophilic, non-toxic gold nanoparticles significantly improved methotrexate delivery into the epidermis and dermis, demonstrating strong potential for nanoparticle-assisted transdermal drug delivery in skin therapy.	2016	132		(28)
Ginseng-berry-mediated gold and silver nanoparticle synthesis and evaluation of their *in vitro* antioxidant, antimicrobial, and cytotoxicity effects on human dermal fibroblast and murine melanoma skin cell lines	Research Article	Jiménez et al.	International Journal of Nanomedicine	South Korea	This study reports the green synthesis of gold and silver nanoparticles using Panax ginseng berry extract and demonstrates their antioxidant, antibacterial, and skin-related biological activities. The gold nanoparticles showed good biocompatibility with skin cells and potential for drug delivery and cosmetic applications, while silver nanoparticles exhibited strong antibacterial and anticancer effects.	2017	114		(29)
Injectable hydrogel for postoperative synergistic photothermal-chemodynamic tumor and anti-infection therapy	Research Article	Huang et al.	Biomaterials	China	This study developed a light-activated injectable nanocomposite hydrogel that combines photothermal and chemo dynamic effects to inhibit tumor recurrence, eliminate multidrug-resistant bacteria, and promote skin wound healing after tumor surgery. The bioactive nanoparticle-based hydrogel enables localized cancer therapy and accelerates skin tissue regeneration, highlighting its potential for post-surgical drug delivery and wound repair.	2022	107		(30)
Gold Nanoparticles Induced Endothelial Leakiness Depends on Particle Size and Endothelial Cell Origin	Research Article	Setyawati et al.	ACS NANO	Singapore	This study demonstrates that gold nanoparticles (10–30 nm) can induce nanoparticle-induced endothelial leakiness (NanoEL), enabling nanomedicine to cross endothelial barriers and access tumors even without the EPR effect. The response depends on endothelial cell type, including sensitivity in human skin endothelial cells, providing guidance for designing gold nanoparticle-based drug delivery systems while managing nanotoxicity.	2017	248		(31)
Near infrared light-triggered on-demand Cur release from Gel-PDA@Cur composite hydrogel for antibacterial wound healing	Research Article	Tao et al.	CHEMICAL ENGINEERING JOURNAL	China	This study introduces a near-infrared responsive nanoparticle microneedle system for skin cancer treatment, integrating chemotherapy and photothermal therapy to enhance efficacy while minimizing side effects. The dissolvable hyaluronic acid microneedles facilitate effective skin penetration and regulated drug delivery, presenting a promising approach for targeted skin cancer treatment.	2021	213		(32)
miR-182 integrates apoptosis, growth, and differentiation programs in glioblastoma	Research Article	Kouri et al.	GENES & DEVELOPMENT	USA	This study demonstrates that miR-182 inhibits glioblastoma progression by modulating tumor growth, apoptosis, and stemness, with its expression correlating to patient survival. Gold nanoparticle-based spherical nucleic acids facilitated the efficient transport of miR-182 across the blood-brain barrier, diminishing tumor burden and enhancing survival, thereby underscoring a promising nanoparticle-mediated drug delivery approach for brain cancer.	2015	192		(33)
Near-infrared responsive 5-fluorouracil and indocyanine green loaded MPEG-PCL nanoparticle integrated with dissolvable microneedle for skin cancer therapy	Research Article	Hao et al.	BIOACTIVE MATERIALS	China	This study reports a near-infrared–responsive nanoparticle-based hydrogel wound dressing that combines antibacterial activity, rapid hemostasis, and good biocompatibility for the treatment of infected skin wounds. The photothermal nanoparticles enable controlled drug release and effective bacterial killing, thereby significantly promoting *in vivo* skin wound healing.	2020	178		(34)
Absorption, distribution, metabolism, and excretion of nanocarriers *in vivo* and their influences	Research Article	Zhang et al.	ADVANCES IN COLLOID AND INTERFACE SCIENCE	Netherlands	This review summarizes the *in vivo* behaviour of nanoparticle nanocarriers, focusing on their absorption, distribution, metabolism, excretion, and drug release mechanisms that limit clinical translation. It highlights how nanoparticle properties and tracing technologies influence safety and effectiveness, providing guidance for designing safer, more efficient drug-delivery nanocarriers.	2020	177		(35)
Recent advances in electrospun polycaprolactone based scaffolds for wound healing and skin bioengineering applications	Review Article	Joseph et al.	MATERIALS TODAY COMMUNICATIONS	Netherlands	This review highlights electrospun polycaprolactone (PCL) scaffolds as promising biomaterials for skin tissue engineering and wound healing due to their extracellular matrix mimicking structure, biocompatibility, and biodegradability. It discusses advances in incorporating bioactive agents and nanomaterials into PCL scaffolds to enhance skin regeneration and promote rapid wound healing, emphasizing the need for clinical translation.	2019	140		(36)
Novel Approach of Using Near-Infrared Responsive PEGylated Gold Nanorod Coated Poly(L-lactide) Microneedles to Enhance the Antitumor Efficiency of Docetaxel-Loaded MPEG-PDLLA Micelles for Treating an A431 Tumor	Research Article	Hao et al.	ACS APPLIED MATERIALS & INTERFACES	China	This study developed a near-infrared–responsive microneedle drug delivery system using PEGylated gold nanorods to combine photothermal therapy and chemotherapy for the treatment of skin tumors. The gold nanoparticle–based microneedles enabled efficient skin penetration, enhanced antitumor efficacy at low drug doses, and achieved complete tumor eradication without recurrence, highlighting strong potential for skin-targeted cancer drug delivery.	2017	139		(37)

Yellow = recognized only in the WOS database, Blue = recognized only in the Scopus database, Green = recognized by both databases.

Most of the most-cited papers come from China, followed by the USA, Egypt, India, and European countries. This shows that China is the leader in research on using gold nanoparticles to deliver drugs through the skin. Publications only in the Scopus database focus on advanced photothermal, photocatalytic, and microneedle-assisted delivery systems. Publications only in WOS, on the other hand, focus on endothelial interactions, nanoparticle biodistribution, and transdermal transport mechanisms. Skin wound healing, antibacterial therapy, and skin cancer treatment are the most common topics covered in the most cited articles. Inflammatory skin disorders and cosmetic-related applications are next. Table 4 shows that the citations are mostly about multifunctional gold nanoparticle platforms that improve skin penetration, control drug release, and have therapeutic effects. This shows how important they are becoming in transdermal and topical drug delivery systems.

The top ten most recent publications retrieved from the WOS and Scopus databases are summarized in Table 5. Most of these highly recent studies were published in 2025, indicating sustained and growing research interest in gold nanoparticles for skin-related drug delivery applications. According to both databases, the selected publications predominantly focus on advanced therapeutic strategies, including photothermal therapy (40, 47), nanocarrier-assisted transdermal delivery (41), microneedle-based systems (45, 50), and nanoformulations for cancer and dermatological treatments (39). Several studies emphasize skin-specific applications such as transdermal biosensing (50), topical corticosteroid delivery (11), wound healing (45, 48), and antimicrobial activity (52), highlighting the relevance of gold nanoparticles in dermatology and tissue regeneration. Review articles addressing the pharmacokinetics, medical importance, and systemic behaviour of gold nanoparticles also appear prominently, reflecting an increasing need to understand their safety and biological performance (43). Overall, the publications listed in Table 5 demonstrate strong interdisciplinary integration of materials science, nanotechnology, and biomedical engineering, underscoring current research trends in gold nanoparticle-based skin drug-delivery systems.

**Table 5:** Top 10 most recent published papers based on Scopus and WOS database.

WOS			Scopus		
Publication title (source)	Year	Reference	Publication title (source)	Year	Reference
Radiation impact on gold-based blood flow and associated heat transfer over a time-dependent stretching sheet influenced by a magnetic dipole *(Journal of Radiation Research and Applied Sciences)*	2025	(38)	Recent advances in improved efficacies of gold nano-formulations in treatment of skin cancer: a systematic review *(Archives of Dermatological Research)*	2025	(39)
Ethosome (ETHOSOMEPT) Photothermal Therapy with Nd:YAG Laser (Pastelle Pro): A Novel Nanotechnology Approach for Treatment-resistant Melasma *(Plastic and Reconstructive Surgery—Global Open)*	2025	(40)	Cold atmospheric plasma for gas therapy and gas-activated drug delivery *(Advanced Drug Delivery Reviews)*	2025	(41)
Medcassoside-functionalized platinum-based liposomes for sensitive skin: Enhancing rapid soothing and barrier homeostasis *(Colloids and Surfaces B: Biointerfaces)*	2026	(42)	Medical importance and pharmacokinetics of gold nanoparticles in the human body *(Molecular Cancer)*	2025	(43)
Cornea-SELEX-derived DNA aptamers for preparing spherical nucleic acids and corneal staining *(Nanoscale)*	2026	(44)	Microneedle-aided nanotherapeutics delivery and nanosensor intervention in advanced tissue regeneration *(Journal of Nanobiotechnology)*	2025	(45)
SERSμDrop: A Platform to Study Cell-Cell Communication via SERS Imaging ***(****Small****)***	2025	(46)	A senescence-responsive nanodrug amplifies radiotherapy efficacy *(Journal of Controlled Release)*	2025	(47)
Tissue engineering with bionanomaterials: A new frontier *(Phytomedicine)*	2025	(48)	Sustainable poly(vinyl alcohol)/chitosan electrospun nanofibers and casted films with bioactive additives—comparative study on physicochemical properties and *in vitro* biological activity (*Industrial Crops and Products)*	2025	(49)
Fenestrated Microneedle Arrays with Hybrid Conductive Ink Coating for Transdermal Biosensing *(Advanced Functional Materials)*	2025	(50)	Nanoparticle-Assisted Surface Patterning of Microneedles for Mechanical Interlocking-Based Tissue Adhesion *(ACS Applied Nano Materials)*	2025	(51)
Medical importance and pharmacokinetics of gold nanoparticles in the human body *(Molecular Cancer)*	2025	(43)	Enhanced antimicrobial and anticancer activities of zein protein-agarose@Au composite hydrogel for controlled release of silibinin in colon cancer therapy *(International Journal of Biological Macromolecules)*	2025	(52)
Topical Nanocarrier-Assisted Corticosteroid Delivery Combined with Phototherapy for Effective Atopic Dermatitis Treatment *(ACS Applied Biomaterials)*	2025	(11)	Next-Gen Cancer Treatment: Nanotechnology-Driven siRNA Delivery Solutions *(Assay and Drug Development Technologies)*	2025	(53)
Osimertinib-Loaded Lactoferrin Nanoparticles for Lung Cancer Therapy *(Bionanoscience)*	2025	(54)	Polymeric microneedles for photothermal therapy *(Proceedings of SPIE—The International Society for Optical Engineering)*	2025	(55)

**Figure 6:**
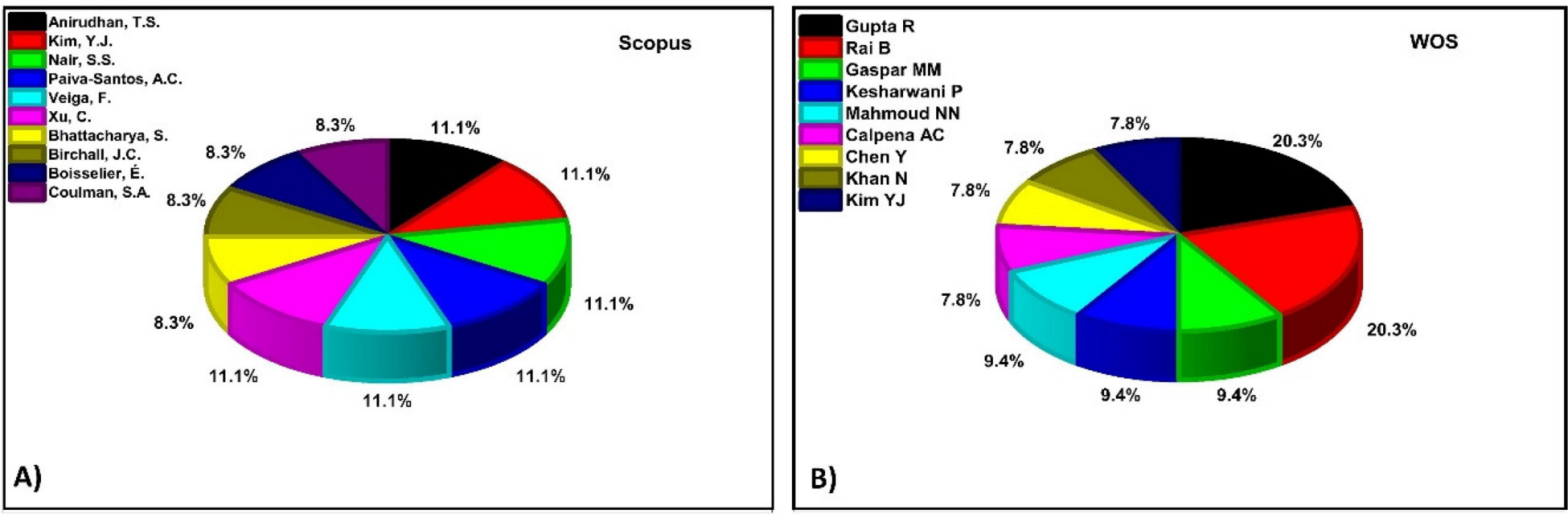
Top 10 authors listed with the highest record of publication based on: **(A)** WOS. **(B)** Scopus.

Figures 6A,B present the top 10 most productive authors in the field of gold nanoparticles in skin drug delivery based on data from the WOS and Scopus databases. According to the WOS database, Gupta R. and Rai B. (22) are the leading authors, each contributing 20.3% of the total publications among the top 10, followed by Gaspar M.M. (56), Kesharwani P. (57), and Mahmoud N.N. (58), each accounting for 9.4%. In contrast, the Scopus database shows a more evenly distributed authorship pattern, with Xu C. (59), Anirudhan T.S., Nair S.S. (60, 61), Kim Y.J. (62), Paiva-Santos A.C., and Veiga F. (63) each contributing 11.1% of the publications. Despite differences in author rankings and contribution percentages, several authors appear across both databases, reflecting variations in database coverage and indexing practices over the study period from 2015 to 2025.

### 3.4 Analysis of keywords

This subsection analyses keyword co-occurrence to uncover major research themes and emerging topics.

**Figure 7:**
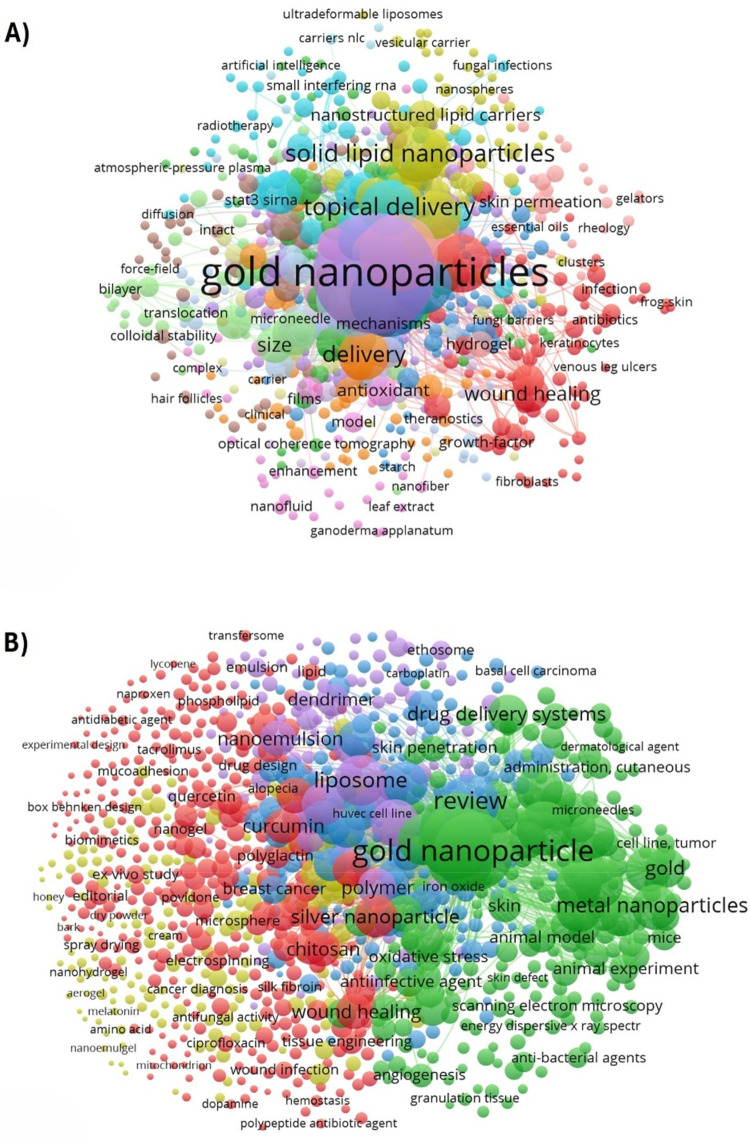
Co-occurrence keywords in the publications based on: **(A)** WOS. **(B)** Scopus.

VOSviewer software was employed to analyse keyword occurrence across publications retrieved from both databases. Keyword data were extracted and imported into the software for analysis. For the WOS dataset, a minimum threshold of two occurrences was applied, and the keyword identification method was set to “All Keywords”, encompassing both Author Keywords and Keywords Plus. Similarly, for the Scopus dataset, a threshold of two occurrences was used, with “All Keywords” including Author Keywords and Indexed Keywords. Full counting was adopted for both datasets. Due to limitations in the WOS database, only the first 500 publications were included in the WOS keyword analysis. Figures 7A,B illustrate the overall keyword co-occurrence networks for the WOS and Scopus databases, respectively, while the five most frequently occurring keywords from both databases are summarized in Table 6. The keywords gold nanoparticles and drug delivery exhibit the highest frequencies in both databases, which is consistent with the initial search criteria. In addition to these common terms, the WOS database highlights keywords such as *in vitro*, nanoparticles, and skin, reflecting a focus on experimental approaches and therapeutic applications. In contrast, the Scopus database prominently includes the terms human, nonhuman, and liposome, which are primarily used to describe experimental models employed in drug delivery-related studies, including nonhuman specimens such as rats and mice. The above thematic clusters reflect key scientific mechanisms underlying gold nanoparticle based drug delivery, including nanoparticle with skin interactions, controlled drug release, and functionalization strategies that improve targeting and stability.

**Table 6:** The top five keywords in the publications based on Scopus and WOS databases.

WOS			Scopus		
Keyword	Occurrences	Total link strength	Keyword	Occurrences	Total link strength
Gold nanoparticles	297	2,760	Gold nanoparticle	391	17,335
*In-vitro*	125	1,233	Drug delivery system	334	15,715
Drug-delivery	119	1,089	Human	317	15,054
Nanoparticles	120	1,058	Nonhuman	274	13,528
Skin	71	731	Liposome	160	8,947

A cluster-based interpretation of the keyword co-occurrence networks (Figures 7A,B) reveals several well-defined thematic groups across both datasets. In Figure 7A, the central cluster is dominated by terms such as gold nanoparticles, topical delivery, and delivery, representing the core focus on transdermal drug delivery mechanisms. A prominent upper cluster is associated with solid lipid nanoparticles and nanostructured lipid carriers, highlighting the development of advanced lipid-based delivery systems. Another cluster is centered around therapeutic applications, particularly wound healing, hydrogel, and growth factors, indicating the increasing focus on tissue repair and regenerative applications. Additionally, emerging terms such as small interfering RNA, radiotherapy, and microneedles suggest the expansion toward advanced and multifunctional therapeutic strategies. In Figure 7B, similar thematic structures are observed, with a central cluster focusing on gold nanoparticles, drug delivery systems, and skin penetration. A distinct cluster related to formulation strategies includes terms such as nanoemulsion, liposome, dendrimer, and polymer, reflecting efforts in optimizing nanoparticle design. Another major cluster represents biological and clinical evaluation, including animal model, tumor, and cutaneous administration. A further cluster is associated with therapeutic outcomes such as wound healing, oxidative stress, and antibacterial activity. This comparison of both datasets demonstrates a consistent progression of the field from fundamental nanoparticle design and formulation toward application-driven research, with increasing emphasis on clinical relevance, advanced delivery systems, and multifunctional therapeutic approaches.

## 4 Conclusion

The bibliometric analysis provides a structured assessment of contemporary trends in global academic literature regarding gold nanoparticle skin drug delivery from 2015 to 2025, utilizing data sourced from the Web of Science and Scopus databases. The results show that the number of publications has been steadily and significantly rising, especially in the last few years. This is because scientists and technologists are becoming more interested in the field.

Most of the research activity is happening in Asia, with India and China being the biggest contributors. This is because of strong national funding bodies and strong institutional output. There are a lot of research articles and review papers, which shows that a lot of people are interested in experimental research and bringing together different kinds of knowledge. But there are not many clinical translations or patent activities, which shows that there are still gaps between research done in the lab and research done in the real world.

Despite significant progress, challenges related to long-term toxicity, biodistribution, and clinical translation remain critical barriers, as reflected by the predominance of *in vitro* and preclinical studies over limited clinical applications. These limitations point to several important research gaps. There is still a lack of standardized methods for nanoparticle synthesis and evaluation, which makes it difficult to compare results across studies. In addition, achieving consistent and controlled drug release under real biological conditions remains a challenge. Future work should focus on additional clinical studies, better standardization of testing methods, and improvements in drug delivery system design. Approaches such as microneedle-assisted delivery, photothermal therapy, and responsive nanocarriers show strong potential for improving treatment outcomes and moving these systems closer to clinical use.

Citation and keyword analysis indicate that key research areas include nanoparticle design, skin penetration, controlled drug delivery, wound healing, antibacterial therapy, and skin cancer treatment, accompanied by increasing attention to photothermal and microneedle-based delivery systems. This study delineates the intellectual framework and progression of the field, identifies significant contributors and nascent topics, and presents critical insights that may inform future research aimed at enhancing clinical translation, safety evaluation, and the development of multifunctional gold nanoparticle-based skin drug delivery systems. Overall, integrating bibliometric trends with scientific advancements shows that the field is evolving from experimental nanoparticle design toward application-driven, clinically oriented research.

## Data Availability

The raw data supporting the conclusions of this article will be made available by the authors, without undue reservation.
